# Discovering Health Topics in Social Media Using Topic Models

**DOI:** 10.1371/journal.pone.0103408

**Published:** 2014-08-01

**Authors:** Michael J. Paul, Mark Dredze

**Affiliations:** 1 Department of Computer Science and Center for Language and Speech Processing, Johns Hopkins University, Baltimore, Maryland, United States of America; 2 Human Language Technology Center of Excellence and Department of Computer Science, Johns Hopkins University, Baltimore, Maryland, United States of America; University of Namur, Belgium

## Abstract

By aggregating self-reported health statuses across millions of users, we seek to characterize the variety of health information discussed in Twitter. We describe a topic modeling framework for discovering health topics in Twitter, a social media website. This is an exploratory approach with the goal of understanding what health topics are commonly discussed in social media. This paper describes in detail a statistical topic model created for this purpose, the Ailment Topic Aspect Model (ATAM), as well as our system for filtering general Twitter data based on health keywords and supervised classification. We show how ATAM and other topic models can automatically infer health topics in 144 million Twitter messages from 2011 to 2013. ATAM discovered 13 coherent clusters of Twitter messages, some of which correlate with seasonal influenza (r = 0.689) and allergies (r = 0.810) temporal surveillance data, as well as exercise (r = .534) and obesity (r = −.631) related geographic survey data in the United States. These results demonstrate that it is possible to automatically discover topics that attain statistically significant correlations with ground truth data, despite using minimal human supervision and no historical data to train the model, in contrast to prior work. Additionally, these results demonstrate that a single general-purpose model can identify many different health topics in social media.

## Introduction

Several studies have utilized social media for tracking trends and analyzing real world events, including news events, [1] natural disasters, [2] user sentiment, [3] and political opinions. [4]–[5] Twitter is an especially compelling source of social media data, with over half a billion user-generated status messages (“tweets”) posted every day, often publicly and easily accessible with streaming tools. [6] By aggregating the words used by millions of people to express what they are doing and thinking, automated systems can approximately infer what is happening around the world. Researchers have begun to tap into social media feeds to monitor and study health issues, [7] with applications in disease surveillance and other epidemiological analysis.

By far the most commonly analyzed disease in social media is influenza. Many researchers have tracked influenza in social media data, most commonly Twitter, using a variety of techniques such as linear regression, [8]–[10] supervised classification, [11]–[12] and social network analysis. [13] Researchers have also used social media to study cholera, [14] dental pain, [15] and cardiac arrest, [16] as well as population behavior including physical activities, [17] mood and mental health, [18]–[19] and alcohol, [9], [20] tobacco, [21] and drug use. [22] Twitter has a desirable property of being a real time data source, in contrast to surveys and surveillance networks that can take weeks or even years to deliver information. Additionally, users of Twitter may candidly share information that they do not provide to their doctor, and thus it is potentially a source of new information, such as off-label use of medications. [23], [24].

Studies like these rely on the *detection* of specific illnesses such as influenza or health topics such as exercise. In this work, we instead describe how to perform *discovery* of ailments and health topics. We do this using topic models, which automatically infer interesting patterns in large text corpora. We believe an exploratory, discovery-driven approach can serve us a useful starting point for medical data mining of social media, by automatically identifying and characterizing the health topics that are prominently discussed on social media. Our goal is not to improve modeling of any one specific illness, but to demonstrate a model for illness discovery. While we may validate the discovered illnesses against specialized approaches for tracking each specific illness, the strength of our model is that it allows discovery of new illness in new data without *a priori* knowledge. Furthermore, our list of discovered illnesses contains several that have previously been unexplored in Twitter, suggesting new areas for directed research, described in the Discussion section.

In this paper, we describe a statistical topic modeling framework for identifying general public health information from millions of health-related tweets. In addition to a basic topic model, we also describe our Ailment Topic Aspect Model (ATAM), previously used to analyze tweets from 2009–10. [24] This framework is used to explore the diversity of health topics that are discussed on Twitter, and we find that many health topics correlate with existing survey data. Our specific contributions are: (1) we describe a current end-to-end framework for data collection and analysis, which includes multiple data streams, keyword filters, and supervised classifiers for identifying relevant data; (2) we analyze a set of 144 million health-related tweets that we have been downloading continuously since August 2011; (3) we provide many previously unpublished details about the creation of our classifier for identifying health tweets and details of ATAM, our specialized health topic model, including procedures for large-scale inference; (4) we evaluate this framework and topic model quality by comparing temporal and geographic trends in the data with external data sources. We experiment with both a basic topic model and ATAM, as well as individual keyword filters for comparison. This article is an extension of an earlier unpublished technical report [25] and includes a longer explanation of ATAM and LDA, more technical detail such as the Gibbs sampling update equations, and more experimental comparisons between various approaches than any of our previous studies on this subject.

## Materials and Methods

### Ethics Statement

The work described in this paper was reviewed by the Homewood Institutional Review Board at Johns Hopkins University and received an exemption since all data is publicly available.

### Data Collection

We used two Twitter datasets from different time periods. The first is a collection of over 2 billion tweets from May 2009 to October 2010. [5] We used this dataset in earlier experiments [24] which were used to inform our current data collection process. The second collection comes from Twitter’s streaming API [26] starting in August 2011 until February 2013, a daily average of 4 million tweets. We select all tweets that match any of 269 health keywords as well as 1% of public tweets. The selection of these 269 keywords was made by identifying words strongly associated with the collection of health-related tweets used in our previous study [24] and manually removing non-informative terms.

We collected 20,000 keyphrases related to illnesses, symptoms, and treatments from two websites. [27]–[28] We added “sick” and “doctor” and removed spurious keywords. These keyphrases were used for our health filter and to identify symptom and treatment words as described below. We selected words from consumer-oriented websites because the language is more likely to match the informal language used in social media as compared to language used in literature intended for medical professionals.

We additionally collected articles concerning 20 health issues from WebMD:[29] allergies, anxiety, asthma, back pain, breast cancer, COPD, depression, diabetes, ear infection, eye health, flu, foot injuries, heartburn, irritable bowel syndrome, migraine, obesity, oral health, skin health, and sleep disorders. As described below, these articles were used to guide model inference. These conditions were selected among the most popular health topics featured on the homepage of WebMD, excluding topics such as sexual conditions that were not commonly discussed health topics in Twitter, based on a preliminary topic model analysis. Within each health condition, we collected all articles that contained information describing the condition and its symptoms and treatments.

### Data Filtering

We filter data to identify health tweets. Keyword filtering, which is used to obtain the data, is insufficient; e.g., “I'm sick of this” and “justin beber ur so cool and i have beber fever.” [8] Instead, we rely on supervised machine learning classification to filter tweets.

We filtered tweets from 2009–2010 with 20,000 keyphrases and randomly annotated a subset of the remaining 11.7 million tweets using Amazon Mechanical Turk, a crowdsourcing service, [30]–[31] to distinguish relevant health tweets from spurious matches. Workers annotated examples as positive (about the user’s health), negative (unrelated, e.g. news updates or advertisements, or not English), or ambiguous. To ensure quality, we annotated a sample ourselves and required workers to annotate some of these “gold” tweets, which allowed us to check annotator accuracy and exclude inaccurate workers. Second, each tweet was labeled by three annotators and the final label was determined by majority vote, removing the 1.1% of examples where the majority vote was ambiguous.

This yielded a set of 5,128 tweets (36.1% positive) for training data to create a classifier for health relevance. We trained a binary logistic regression model using the MALLET toolkit [32] with n-gram (1≤n≤3) word features. We tokenized the raw text such that contiguous blocks of punctuation were treated as word separators, with punctuation blocks retained as word tokens. We removed tweets containing URLs, which were almost always false positives.

We tuned the prediction threshold using 10-fold cross validation to result in an estimated 68% precision and 72% recall, a balance of precision and recall. Applying this classifier to the health stream yielded 144 million health tweets, a nearly hundred-fold increase over our earlier study of 1.6 million tweets. [24].

#### Location Filtering

For experiments that require geographic information, we used Carmen, a Twitter geolocation system. [33] Carmen relies on a combination of GPS coordinates from mobile devices and user-supplied profile information (e.g. “NYC”, “The Big Apple”) to determine the location (city, county, state, country) associated with each tweet, when possible.

### Model Descriptions

Our approach to identifying health topics is based on the framework of probabilistic topic modeling [34] for text analysis. We describe two such topic models.

#### Latent Dirichlet Allocation (LDA)

Latent Dirichlet Allocation (LDA) [35] assumes that a text document has some probability distribution over “topics,” and each such topic is associated with a distribution over words. Topics are not observed as input, rather they are inferred. Topic models are unsupervised models; they can be thought of as automatically clustering words into topics and associating documents with those topics.

LDA posits that each word (token) *n* in a document *d* has a variable *w_dn_* that represents the observed word type (i.e. a dictionary entry) as well as a latent topic variable *z_dn_*. Under this model, a word token is generated by randomly sampling a value *z_dn_ = k* from the document’s topic distribution *θ_d_*, then sampling a word type *w_dn_ = v* from the topic *k*’s word distribution *φ_k_*. Given the parameters *θ* and *φ*, the marginal probability of a word under the LDA model is: 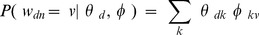.

Each word is conditionally independent given the parameters. LDA is a Bayesian model in which there are also distributions (priors) over the parameters *θ* and *φ*, given by Dirichlet distributions with hyperparameters *α* and *β*.

In our experiments, we use a variant of LDA that includes an additional “background” word distribution to model common, non-topical words, which can produce less noisy topics. [36]–[37] This model assumes that each word is generated under the standard LDA model with probability *λ*, while with probability 1*–λ* the word comes from the background distribution. This concept is also in ATAM, described below.

#### Ailment Topic Aspect Model (ATAM)

Preliminary LDA experiments discovered health-related topics around ailments but many other topics as well. For example, some topic clusters would correspond to symptom terms that could be associated with many illnesses.

Consider the example sentence, “damn flu, home with a fever watching TV.” It contains two words relevant to the ailment of flu (“flu,” “fever”), one of which is a symptom. It also contains words that are not about the ailment but are topically related (“home,” “watching,” “TV”), which might be described by a “stay at home” topic. Finally, it contains common words that would not be described with a particular topic or ailment (“damn,” “with,” “a”).

We developed a model that explicitly labels each tweet with an ailment category and distinguishes ailment words from other topics and non-topical words. Our model includes a standard LDA model to explain non-ailment topics, but also includes a model to filter out background noise and a specialized ailment model that incorporates symptom and treatment information.

Under our model, each tweet *d* is categorized with an ailment *a_d_ = i* with probability *η_i_*. Each word token *n* in tweet *d* is associated with two observed variables: the word type *w_dn_*, and a label *y_dn_* that we call the “aspect” which denotes whether the word is a symptom word, treatment word, or anything else – a general word. The *y* variables are given as input; the dataset is labeled using the list of 20,000 symptom and treatment keyphrases described above. Each word token in a tweet is generated as follows.

- **Background model:** The word is assumed to be background noise (binary random variable *ℓ_dn_*) with probability 1*–λ*, and it is a non-background word with probability *λ*. If the word *w_dn_ = v* is background noise, it has probability *φ_B,jv_*, where *y_dn_ = j*. The background word distributions are shared across the entire dataset and each aspect has a separate distribution.
- **Topic model:** Non-background words are either an ailment word with probability *π_d_* or a non-ailment topic word with probability 1*–π_d_* (binary random variable *x_dn_*). If it is a topic word, then the word’s probability is given by the standard LDA model: the word is associated with topic *z_di_ = k* with probability *θ_dk_*, and the topic *k* generates the word *w_dn_ = v* with probability *φ_T,kv_*. Each topic has its own word distribution.
- **Ailment model:** If the word is an ailment word, then the word probability depends on both the tweet’s ailment label and the token’s aspect label. The ailment *a_d_* = *i* generates the word *w_dn_ = v* with probability *φ_A,ijv_*, where *y_dn_ = j*. Each ailment has three separate word distributions for general words, symptom words, and treatment words. The distributions of ailment words is thus structurally different from the distributions of topic words, which do not distinguish symptom and treatment words from others.

Having separate word distributions for each aspect is an idea borrowed from the Topic Aspect Model (TAM), [38] in which topics in a topic model are decomposed into multiple aspects (similar to “cross-collection” [39]–[40] or “multi-view” [41] topic models). We thus call our model the Ailment Topic Aspect Model (ATAM). Conditioned on the parameters and the ailment *a_d_ = i*, the likelihood of a word token *w_di_* under ATAM is:
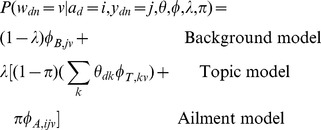



As in LDA, we place Dirichlet priors over the model parameters. These prior probabilities are formulated as follows.

- **Word priors:** We place informative priors over the word distributions to incorporate knowledge from external resources into the model: in this case, a Dirichlet distribution centered around the word distribution found in the WebMD articles. Specifically, for the ailment *i* and each aspect *j*, *φ_A,ij_* is distributed according to Dirichlet (*β_i_*), where *β_i_*  = s_i_*m_i_ such that *m_i_* is a vector of the empirical unigram word distribution in the WebMD articles pertaining to the *i*th ailment, and *s_i_* is a scalar precision parameter. This encodes an *a priori* belief that the ailment word distributions are likely to match the word distributions in these health and medical articles. The precision *s* controls the degree of this belief and can be automatically adjusted to optimize marginal likelihood. We fix *β* = 0.01 for the non-ailment distributions.
- **Topic priors:** Each document’s topic distribution *θ_d_* has a Dirichlet (*α_i_*) prior, where the document ailment variable *a_d_ = i*. That is, there is a separate *α_i_* vector for each ailment value, so the document’s prior over topic distributions depends on the document ailment. This allows the model to make associations between particular ailments and particular non-ailment topics.
- **Other priors:** The other parameters all have simple symmetric and pre-specified Dirichlet or Beta (the bivariate analog) priors, which act as regularizers: *η*∼Dirichlet (*σ*), *π_d_*∼Beta (*γ*), both set to 1.0 in our experiments. We do not place a prior over the background noise parameter *λ*; instead we assume this parameter is given as input to control the degree of noise filtering, set to 0.2 in our experiments (i.e. probability of noise is 0.8).

**Figure 1:**
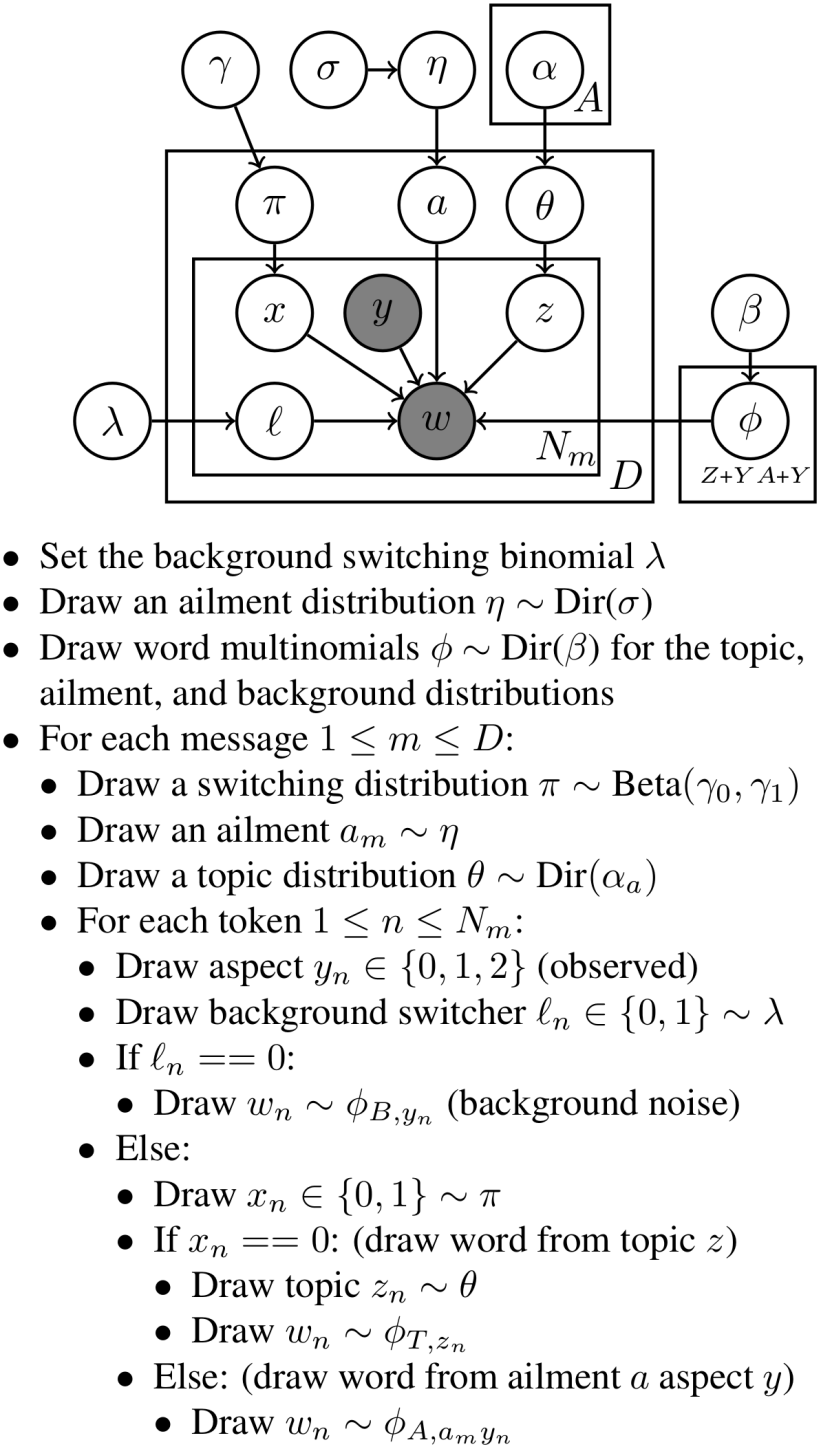
The graphical model and generative story for ATAM. The graphical model representation of ATAM using plate notation, followed by the “generative story” description of the model. In the graphical model, the variable *z* denotes the topic index, and the Bernoulli variables *x* and *ℓ* are switch variables indicating whether a word is an ailment or topic word and whether a word is background noise. These three variables do not appear in the conditional likelihood because they have been summed out. *A* is the number of ailments, *Y* is the number of aspects, *Z* is the number of topics, *D* is the number of documents, and *N_m_* is the number of tokens in document *m*. In the generative description, “Dir” refers to the Dirichlet distribution.

The marginal likelihood of the data under these priors is:
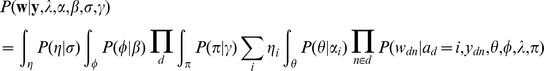



Figure 1 shows the graphical model representation of ATAM along with its probabilistic “generative story”.

In our experiments, we fixed both the number of ailments and the number of topics to 20.

### Model Inference

#### Posterior Inference

ATAM includes many variables and parameters which must be inferred. Our goal is posterior inference, the standard type of inference used in LDA-based models, [42] in which we infer a distribution over the parameters. A popular method of posterior inference in topic models is Gibbs sampling, [43] a Markov Chain Monte Carlo method. [44] In a Gibbs sampler, values of each variable will be sampled according to the posterior distribution, and with enough samples, the expected value of the variable can be reasonably approximated. The algorithm iteratively samples a new value for each random variable from the conditional distribution given the current values of all other variables. We can derive a *collapsed* Gibbs sampler by marginalizing the multinomial parameters out of the sampling equations, requiring us to only sample the variables *a*, *z*, *x* and *ℓ*. [43] We alternately sample the document-level variable *a* and the token-level variables (*z*, *x*, *ℓ*). The sampling equations for these four variables are given at the end of this section. We ran the Gibbs sampler for 8000 iterations. We use the same inference procedure for LDA. [43], [36].

#### Hyperparameter Optimization

The Dirichlet hyperparameters *α* and *β* are optimized during the inference procedure. We alternate between running the Gibbs sampler conditioned on the current hyperparameters for 10 iterations, then optimizing the hyperparameters to maximize the marginal likelihood of the sampled variables. We use the fixed-point iterative update equations derived by Minka [45] to optimize the hyperparameters of a Dirichlet-multinomial distribution. Recall that for ATAM’s priors over word distributions, we have defined each *β_k_* such that the precision *s_i_* and mean *m_i_* are decoupled. In this case, the mean is fixed, and we only update the precision *s_i_*. For the priors over topic distributions, we freely optimize each *α_i_* without such constraints. Minka provides update equations for both scenarios.

#### Large Scale Inference

We relied on two procedures to handle our large dataset. First, we use an iterative map-reduce framework to distribute the computation. [46] Gibbs samplers are independently run on different shards of data (map stage), and at the end of the sampling iteration, the counts across all shards are pooled together and the sufficient statistics within each process are updated to reflect the current global counts (reduce stage). We ran our distributed ATAM and LDA implementations across 50 processors.

Second, we initially ran the sampler on smaller subsets of data and incrementally brought in more data, under the intuition that the inference algorithm may learn good parameters on a smaller sample of the data. Our implementation fed data to the sampler in 10% increments. Each time additional data is added, the variables are initialized to their optimal value under the current sampler state. The increment schedule is that a fraction *t* of the data is sampled for 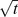 of the iterations, so more iterations are spent on less data.

#### Gibbs Sampling Equations for ATAM

Assignments to ailments *a* are sampled for each document according to the following distribution:
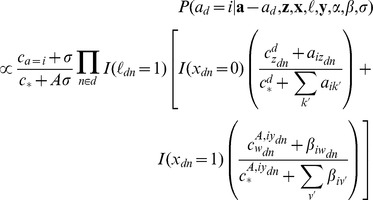



Assignments to *ℓ, x*, and *z* are sampled for each token according to the following distributions:



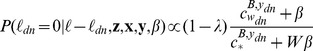
.
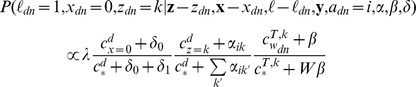


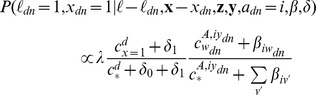



The notation ***a*** *–a_d_* denotes the set of a variables excluding *a_d_*, as the sampling distribution is conditioned on all variables except for the one being sampled. The *c* variable denotes sufficient statistics of the current sampler state; specifically, 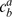 denotes the number of times *b* appears in *a*, with * being a wildcard. For example, 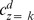 is the number of times the topic variable *z* was assigned to value *k* in document *d*. *A* is the number of ailments, *Z* is the number of topics, and *W* is the size of the vocabulary. *I*(*x*) is an indicator function that returns 1 if the expression *x* is true and 0 otherwise.

### Mining Trends

Our goal is to discover coherent ailments composed of groups of tweets. While we will present an analysis directly on these groups, we also seek extrinsic validation of these groups by utilizing them for various tasks. We do not expect to outperform methods crafted specifically for these tasks, rather we use them to measure whether our unsupervised approach has discovered a signal of interest.

For extrinsic evaluations, we consider two types of analysis based on the ailments: the prevalence of ailments over time and over geographic regions. For an ailment *i*, we consider P(*a = i* | time period) or P(*a = i* | region), computed as the percentage of tweets assigned to ailment *i* for that time period or region. We do the same with LDA, with topics instead of ailments.

In our experiments, we also consider trends of individual keywords for comparison, in which case we simply count the number of tweets containing a keyword, normalized by the total number of tweets in the dataset from that time period or region.

### Temporal Trends

We consider two ailments with seasonal temporal patterns: influenza and allergies. While there is a body of work on tracking influenza on Twitter, [7] the surveillance of allergy symptoms is a novel use of Twitter. We do not use geolocation in these experiments.

#### Influenza over Time

We computed the Pearson correlation between the weekly influenza rate in Twitter, as measured using the topic model ailment most closely resembling influenza, and weekly data from the Centers for Disease Control and Prevention (CDC). In particular, we use data from the U.S. Outpatient Influenza-like Illness Surveillance Network (ILINet), [47] which measures the percentage of outpatient visits due to influenza-like illness in the United States.

Our data spans two influenza seasons. The 2011–2012 season began October 2, 2011 (n = 52 weeks). The 2012–2013 season began September 30, 2012. Our results for the 2012–2013 season only go up to the week beginning February 24, 2013, which was the last week of data in our Twitter collection (n = 22).

#### Allergies over Time

We computed the Pearson correlation between the monthly allergy rate in Twitter and monthly survey data given by a Gallup-Healthways poll. This data gives the percentage of respondents who answered yes to the question, “Were you sick with allergies yesterday?” in telephone interviews of adults in the United States. The survey data includes monthly rates from 2010 through April 2012. [48].

Our dataset overlaps the survey data from August 2011–April 2012 (n = 9). Additionally, we also compared all of our Twitter data from August 2011–February 2013 (n = 19) to Gallup data, where after April 2012 we use each month’s data from the previous year, under the assumption that the monthly trend is similar across years. This allowed us to compare all months of our Twitter data to approximate survey data.

Our earlier conference paper gave examples of allergy trends but did not compare to external data. [24] To the best of our knowledge, this is the first time Twitter data has been compared to external survey data about allergies.

### Geographic Trends

To evaluate geographic trends, we measured the Pearson correlation between the ailment rates in U.S. states (n = 51, including the District of Columbia) with survey data for various health and lifestyle factors such as physical activity. We used survey data from the CDC’s Behavioral Risk Factor Surveillance System (BRFSS), which includes survey results from phone interviews of over 500,000 adults in the U.S. in 2011. [49] This important large-scale survey provides a single source of data for a variety of experimental comparisons. We measured the correlation between the “diet and exercise” ailment/topic with the following four BRFSS results which are associated with dietary and exercise patterns: the percentage who participated in physical activity and aerobic exercise, the percentage who are obese (BMI > = 30.0), the percentage who have been diagnosed with diabetes (data from 2010; not asked in 2011), and the percentage who have high cholesterol. We also measured the correlation of the “cancer and serious illness” ailment with the following three related BRFSS results: the percentage who have or have had cancer, the percentage who have had a heart attack, the percentage who have had heart disease, and the percentage who have used tobacco. A subset of these factors were also considered in our conference paper using 2009–2010 data. [24].

## Results and Discussion

### Ailment Discovery

**Figure 2:**
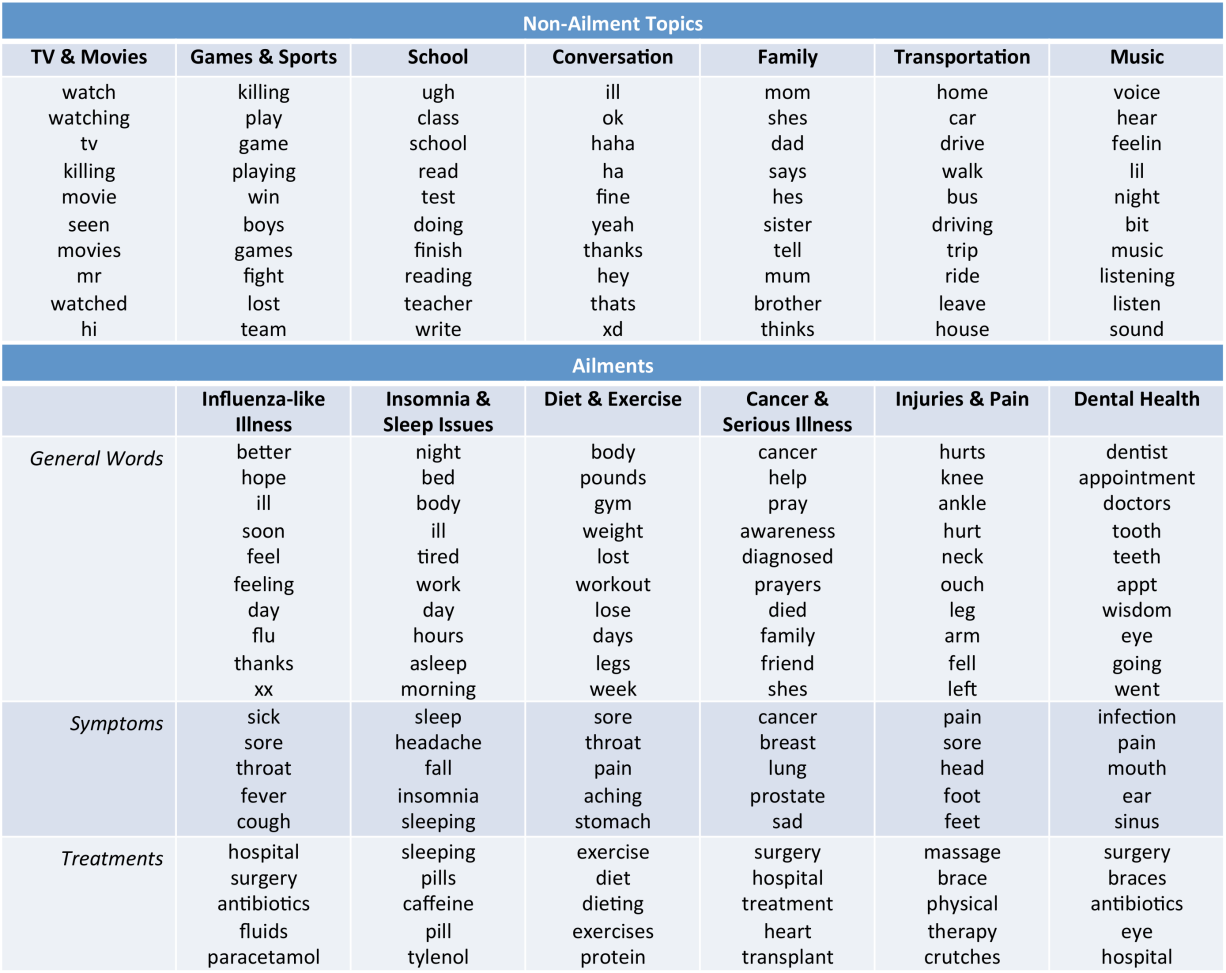
Top words associated with ailments and topics. The highest probability words for a sample of ailments and non-ailment topics. The top ten general words are shown for ailments along with the top five symptom and top five treatment words. The top ten words are shown for topics. The names of the ailments and topics are manually assigned by humans upon inspection of the associated words.

Figure 2 shows examples of the most probable words for various ailments as well as non-ailment topics in ATAM. In addition to the six ailments shown in the table, we identified the following: allergies, depression, cough and respiratory illness, anxiety, sports injuries, hunger and stomach pain, and body image and skin health (13 total). These designations are manually assigned based on the coherence of the most probable words. We note that the model parameters include only unigram word distributions and words can appear as different aspects depending on the larger context. For example, “eye” would be counted as a symptom word if it is part of the phrase “red eye”, a treatment word if part of the phrase “eye drops”, and a general word if not part of a symptom/treatment phrase. As is usually the case with unsupervised topic models, many of the word clusters lacked semantic coherence, [50] and we did not consider incoherent ailment clusters in analysis. Even the coherent ailment clusters exhibit some noise, such as “throat” in the diet and exercise cluster, which is because this word commonly co-occurs with “sore” which is a top symptom word in this cluster (as in “legs are sore”). This is a drawback of unigram word models, but our quantitative experiments below show that these clusters are still capturing meaningful concepts.

### Topic Coherence

Our intrinsic evaluation of ATAM is based on a user study comparing the quality and interpretability of ATAM to LDA. Our goal is to directly evaluate the coherence of ATAM ailments. We performed experiments using Amazon’s Mechanical Turk, a crowdsourcing system, on the Twitter dataset from 2009–10. We labeled the resulting topics so that they could be aligned across the two models for comparison. Three annotators (the second author of this paper and two computer science graduate students) each labeled the resulting LDA topics and ATAM ailments with either an ailment name or as “non-ailment” and we then obtained a consensus as to the best label for each topic/ailment. These experiments are described in our earlier technical report. [25].

We then evaluated model output through two Mechanical Turk experiments. First, we measured agreement of annotators on labeling clusters (ailments/topics). We displayed the top 8 general words, 5 symptoms and 5 treatments for each cluster. Symptom and treatment words were identified in LDA by separating out those words appearing in the keyphrase lists as a post-processing step. We then showed three randomly sorted ailment names (one correct and two randomly chosen) as well as “other” and “junk” options. 80 annotators provided annotations. ATAM discovered more ailments as measured by the number of ailments agreed to by two thirds of the annotators; 14 unique ATAM ailments versus 10 for LDA. Additionally, ATAM produced more identifiable ailments; 45% of annotators agreed with our consensus LDA labels versus 70% for ATAM.

We next sought to evaluate which model produced more coherent ailment clusters. Using our labels, we paired ATAM and LDA clusters that represented the same ailment (e.g., both were labeled as influenza). We then displayed each ailment as before, but now side by side, randomly permuting which appeared on which side, with the ailment name. 67 annotators were asked to select the list of words (including symptoms/treatments) that best described the given ailment, or to indicate a tie otherwise. ATAM was favored over LDA in 11 out of 18 comparisons with an average of 55% of the votes (median 64%).

These experiments show that ATAM discovers more human-identifiable ailments with higher coherence than LDA.

### Temporal Trends

#### Influenza

**Figure 3:**
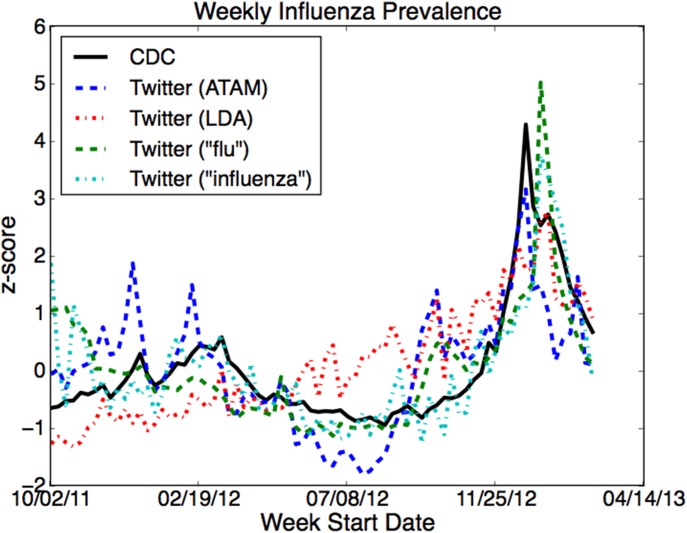
Influenza over time. The weekly rate of influenza as estimated by the volume of tweets assigned to the influenza-like illness topics and keywords alongside the rates given by the CDC ILINet (solid black line). The better of the two LDA topics is shown. All rates are standardized (z-scores) so that they are comparable on the y-axis.

The weekly rate P(*a*) for the ATAM ailment we identified as “influenza-like illness” correlated strongly with the CDC ILI data. These correlations are shown in Table 1. Figure 3 shows the CDC and Twitter trends over time. We observe that the ATAM trend has lower variance and the rate does not fall in off-season weeks as much as the CDC data. This may be because there is background noise grouped with the influenza ailment on Twitter, so the baseline rate is high. Nevertheless, the rates from the data sources often peak in the same week, and the Twitter rate in 2012–2013 is higher than 2011–2012, in agreement with the ground truth trend.

**Table 1:** Pearson correlations between various Twitter models and keywords and CDC influenza-like illness (ILI) surveillance data for three time periods.

	2011–12	2012–13	2011–13
ATAM	.613	.643	.689
LDA (1)	.670	.198	.455
LDA (2)	−0.421	.698	.637
“flu”	.259	.652	.717
“influenza”	.509	.767	.782

The two LDA rows correspond to two different LDA topics.

LDA discovered two topics that contain ILI-related words. The first is very similar to the ATAM ILI ailment. The second has “fever” as the top two word, with “flu” among the top ten, but the rest of the word distribution is noisy. ATAM is significantly more correlated with both seasons than the first LDA topic (p≤0.034) and the second LDA topic in the second season (p<0.001). The difference between ATAM and the second LDA topic are not significant across both seasons.

Two individual keywords, “flu” and “influenza” have higher correlations in the later two seasons than the topic models though the differences are all insignificant (p≥0.222). ATAM is significantly better than “flu” in the 2011–12 season (p = 0.026) but not “influenza” (p = 0.453). Since topic models combine many keywords to determine a tweet’s relevance to influenza, we are encouraged by its ability to discover these word groups such that they obtain levels similar to hand-picked keywords.

#### Allergies

**Figure 4:**
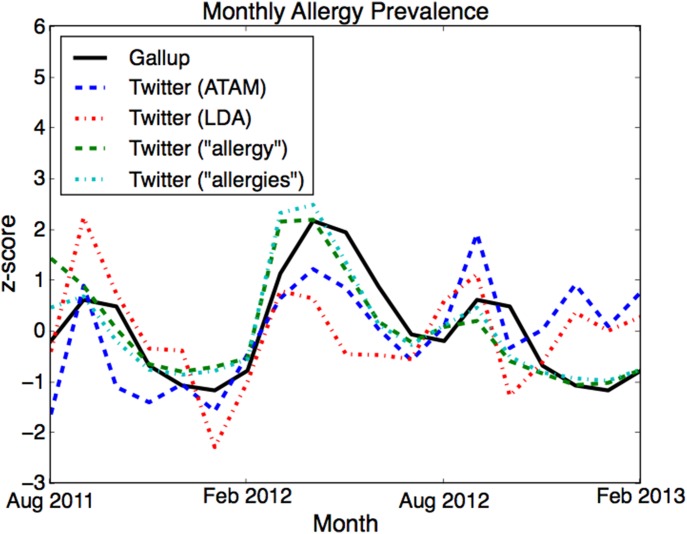
Allergies over time. The monthly rate of influenza as estimated by the volume of tweets assigned to the allergies topics and keywords alongside the rates given by the Gallup phone survey (solid black line). Gallup data after April 2012 does not exist, so we duplicated the same rates from the previous year (05/2011–02/2012). All rates are standardized (z-scores) so that they are comparable on the y-axis.

We selected the ailment we identified as “allergies and colds” for the allergies experiments. Correlation results are shown in Table 2. Figure 4 shows the Gallup and Twitter trends over time. As with the influenza plot, there is less variance in the Twitter curve than the survey data. However, all of the spikes line up, with one exception: in December of 2011 and 2012, there was a small spike of the ATAM rate that is not present in the survey data. We believe this is because the common cold is mixed in with this ATAM ailment, and cold-related messages increase in the winter. This spurious rise is stronger in 2012 and persists through 2013, which may be due to the unusually strong influenza season this year, [47] during which people report similar symptoms.

**Table 2:** Pearson correlations between various Twitter models and keywords and Gallup allergy survey data for two time periods.

	08/11–04/12	08/11–02/13
ATAM	.810	.479
LDA	.705	.366
“allergy”	.873	.823
“allergies”	.922	.877

The earlier period is the original data, while the data after April 2012 is from the previous year (05/2011–02/2012).

LDA discovered a similar allergy-related topic, but this topic also contained noise from similar symptoms for other ailments, which were less correlated than ATAM. The differences between ATAM and LDA correlations were not significant. Across all months, the keyword “allergies” has a significantly higher correlation than the topic models (p≤0.012), with no significant differences from the topic models in the earlier time period. The keyword “allergy” had slightly weaker correlations than “allergies”.

### Geographic Trends

The ATAM ailment we identified as “diet and exercise” is significantly and often strongly correlated (p≤0.001) with all pertinent BRFSS statistics. LDA’s similar diet and exercise topic as well as the “diet” and “exercise” keywords all have very similar correlations which are not significantly different. These correlations are shown in Table 3.

**Table 3:** Pearson correlations between various Twitter models and keywords and CDC BRFSS data for various diet and exercise risk factors.

	Activity	Exercise	Obesity	Diabetes	Cholesterol
ATAM	.606	.534	−.631	−.583	−.194
LDA	.518	.521	−.532	−.560	−.146
“diet”	.546	.547	−.567	−.579	−.214
“exercise”	.517	.539	−.505	−.611	−.170

The ATAM ailment we identified as “cancer and serious illness” is not strongly correlated with any pertinent BRFSS statistic. The corresponding LDA topic and keywords “cancer” and “surgery” are similarly weak. Some of the keyword correlations are stronger (up to magnitude of 0.23, p<0.001) but these are still relatively weak and not necessarily in the direction one would expect (“cancer” has negative rather than positive correlations with related risk factors). One possible explanation for these weak correlations is that most tweets in this ailment group appear to be describing friends and family rather than the user personally, so the incidents described might actually occur in other locations. Another explanation is that tweets in this group may be promoting awareness rather than reporting incidence, which could perhaps also explain the reversed direction of the correlation. These correlations are shown in Table 4.

**Table 4:** Pearson correlations between various Twitter models and keywords and CDC BRFSS data for various serious illness risk factors.

	Cancer	Tobacco	Heart Disease	Heart Attack
ATAM	.030	.069	.043	.080
LDA	−.045	−.005	−.069	−.023
“cancer”	−.037	−.180	−.232	−.181
“surgery”	−.049	.188	.021	.060

## Discussion

These results show that topic models can discover a number of ailments that are significantly and often strongly correlated with ground truth surveillance and survey data. Surprisingly, in contrast to prior work that trained systems to identify specific diseases, these trends were identified without human supervision or historical survey or surveillance data. Instead, the unsupervised models automatically discovered word clusters that meaningfully correspond with real world events, which suggests that topic models could discover novel ailments and trends. This is a critical point: even though keyword-based or supervised methods may yield better correlations on specific tasks, it is impressive that general-purpose topic models can discover similar information across numerous ailments. This suggests that topic models can be adapted to find topics on novel health data sets, such as specialized online communities, [22] and because the models require minimal input, there is even potential for the discovery of novel ailments, such as during a disease outbreak.

Beyond using standard topic models, we created ATAM specifically for the purpose of modeling health topics, in line with other research creating specialized topic models for analyzing medical text. [51]–[52] Moreover, we showed a simple way to incorporate domain knowledge via word priors created from external resources. By creating an example of how to create a specialized model augmented with prior knowledge, we hope that medical domain experts can contribute in future work to craft topic models that are more appropriate for specific tasks than off-the-shelf tools. While LDA and ATAM did not have significantly different results in some experiments, ATAM performed better at influenza detection and was shown in a user study to have more interpretable clusters. The addition of informative word priors was also shown in our earlier work to result in ailment clusters that more closely correspond to specific ailment categories. [24].

Our work differs from previous social media based public health analyses in that our aim was broad rather than deep. Rather than focusing on a particular health issue, our purpose was exploratory, and we identified multiple health issues. We conducted a large scale analysis with over a hundred million tweets to identify numerous health trends. Our results show that indeed many different ailments and health issues are discussed on Twitter beyond what has been commonly studied, such as influenza. For example, injuries, stomach pain, and skin health have not been analyzed in depth in Twitter, to our knowledge. Behavioral topics, such as diet and exercise patterns, have also been understudied in social media, especially in light of their importance to behavioral medicine. [53] Our model’s characterization of these ailments and their associated keywords could serve as a helpful starting point for deeper analysis of each ailment in the future.

While individual keywords were often as good as or better than the topic models in our experiments, the topic models can help with keyword identification, particularly for less obvious words that are used on Twitter, and can automatically organize many words into a small number of topics. Topic models also have the advantage of capturing co-occurrences of words within tweets. For example, the influenza ailment includes words like “hope”, “feel”, and “better”, which in the context of influenza are highly indicative of a person experiencing the illness rather than talking about it in non-experiential contexts that might get captured by the keyword “flu” alone. This property may make ATAM more robust and could explain why this model did better than individual keywords in the 2011–12 influenza season, which was mild and difficult to capture. [12] Finally, we note that the keyword baselines are applied to the subset of tweets that our classifiers had already identified as relevant, removing many spurious matches that likely would have worsened the results if we had applied the simple keyword filters to the full set of tweets.

There are inherent limitations in using Twitter and other social media websites for health analyses. Many people will not publicly share their health statuses online, and Twitter is not a representative sample of the population. However, we have shown that a variety of trends can be detected despite these limitations, and it has been shown that such analyses can be adjusted to account for demographic biases. [54] While far from perfect, we believe social media sources can complement existing surveillance tools, with some unique advantages such as near real-time access to naturalistic information.
